# *N*-Acetylaspartate reductions in brain injury: impact on post-injury neuroenergetics, lipid synthesis, and protein acetylation

**DOI:** 10.3389/fnene.2013.00011

**Published:** 2013-12-26

**Authors:** John R. Moffett, Peethambaran Arun, Prasanth S. Ariyannur, Aryan M. A. Namboodiri

**Affiliations:** Neuroscience Program, Department of Anatomy, Physiology and Genetics, Uniformed Services University of the Health SciencesBethesda, MD, USA

**Keywords:** NAA, acetyl coenzyme A, aspartoacylase, acetyl coenzyme A synthase, mitochondria

## Abstract

*N*-Acetylaspartate (NAA) is employed as a non-invasive marker for neuronal health using proton magnetic resonance spectroscopy (MRS). This utility is afforded by the fact that NAA is one of the most concentrated brain metabolites and that it produces the largest peak in MRS scans of the healthy human brain. NAA levels in the brain are reduced proportionately to the degree of tissue damage after traumatic brain injury (TBI) and the reductions parallel the reductions in ATP levels. Because NAA is the most concentrated acetylated metabolite in the brain, we have hypothesized that NAA acts in part as an extensive reservoir of acetate for acetyl coenzyme A synthesis. Therefore, the loss of NAA after TBI impairs acetyl coenzyme A dependent functions including energy derivation, lipid synthesis, and protein acetylation reactions in distinct ways in different cell populations. The enzymes involved in synthesizing and metabolizing NAA are predominantly expressed in neurons and oligodendrocytes, respectively, and therefore some proportion of NAA must be transferred between cell types before the acetate can be liberated, converted to acetyl coenzyme A and utilized. Studies have indicated that glucose metabolism in neurons is reduced, but that acetate metabolism in astrocytes is increased following TBI, possibly reflecting an increased role for non-glucose energy sources in response to injury. NAA can provide additional acetate for intercellular metabolite trafficking to maintain acetyl CoA levels after injury. Here we explore changes in NAA, acetate, and acetyl coenzyme A metabolism in response to brain injury.

## INTRODUCTION

*N*-acetylaspartate (NAA) is one of the most abundant brain metabolites and is highly concentrated in neurons, but it remains to be determined why neurons synthesize so much of this particular acetylated amino acid. Early research implicated NAA in lipid synthesis in the brain, especially during postnatal myelination. Other early investigations connected NAA to carbon transport and energy metabolism in neuronal mitochondria. Subsequently it was discovered that mutations in the gene for the enzyme that deacetylates NAA, known as aspartoacylase or ASPA, lead to the fatal neurodegenerative disorder known as Canavan disease. After more than two decades of research the precise connection between the inability to deacetylate NAA and the pathogenesis of Canavan disease remains a matter of ongoing investigation. One line of research has focused on the lack of catabolism leading to a toxic buildup of NAA in the brain as the primary etiological component. Another line of research has suggested that the lack of catabolism results in an acetate deficiency in oligodendrocytes during brain development that subsequently limits acetyl coenzyme A (acetyl CoA) availability during this critical period of myelination. There is experimental support for both mechanisms, and it is possible that both are operative.

Continuing work on NAA over the last two decades has focused almost exclusively on the utility of NAA measurements in various neuropathological conditions using proton magnetic resonance spectroscopy (MRS). NAA provides the largest single peak on proton MRS spectra of the healthy human brain, and brain NAA levels are found to be reduced in a majority of neuropathologies including traumatic brain injury (TBI). The loss of NAA after TBI is paralleled by a loss of ATP, acetyl CoA, and other metabolites associated with energy metabolism (Vagnozzi et al., 2007) indicating a substantial impact on neuroenergetics. The connections between NAA and brain energy metabolism are not entirely clear, but it seems likely that they involve acetyl CoA generation and utilization (Ariyannur et al., 2010a). Acetyl CoA is at the crossroads of energy derivation, storage, and utilization, and is also involved in cellular control of protein function and gene transcription through acetylation reactions. NAA is synthesized from acetyl CoA and aspartate, and because of the exceptionally high concentration in the human brain (~10 mM) some proportion of acetyl CoA must be utilized to maintain NAA levels, and that proportion may change with brain injury. Signoretti and colleagues have shown that severe brain injury results in a very rapid drop in NAA levels that is paralleled by a similar reduction in ATP levels, suggesting that NAA is utilized rapidly in response to injury. Indeed, NAA levels are reduced by over 20% within 1 min of severe TBI in rats at a time when ATP levels are only reduced by ~10% (Signoretti et al., 2001). These investigators also found that over time after injury, recovery of NAA levels “has been observed only in concert with restoration of ATP” (p. 988) further linking NAA with post-injury neuroenergetics. As such, the study of NAA metabolism may provide unique insights into how the brain’s energy systems respond to injury and recover throughout the healing process.

### NAA SYNTHESIS AND LOCALIZATION

Early investigations into the amino acid content of protein-free brain extracts found a large proportion of “bound aspartic acid,” which represented the largest quantity of any conjugated amino acid present in the brain (Tallan et al., 1954). Because the aspartic acid conjugate was ninhydrin-negative it was concluded that it was N-substituted. Subsequent work demonstrated that the compound was NAA (Tallan et al., 1956). Early studies on the incorporation of ^14^C-labeled glucose into NAA in the adult cat brain suggested low turnover rates, and these findings were interpreted to mean that NAA was metabolically inert (Margolis et al., 1960). Nonetheless the high concentration of NAA in the brain led a small number of researchers to begin investigating how NAA was synthesized in the nervous system.

### ASPARTATE *N*-ACETYLTRANSFERASE

The earliest studies on NAA synthesis indicated that an enzyme was present in the soluble fraction of brain homogenates with an absolute specificity for acetyl CoA and aspartate (Goldstein, 1959). This same study reported that a mechanism existed for the hydrolysis of NAA in the brain, as most of the NAA in rat brain supernatants was lost on 2–3 h incubations at 37°C. These initial findings on NAA synthesis in brain homogenates were called into question by Jacobson (1959), who only detected NAA synthesis in minced brain preparations, but not in cell-free preparations. Jacobson also found that the incorporation of radiolabeled acetate into NAA was highest during brain development while myelination was taking place. This issue was revisited by Knizley (1967) who observed NAA synthesis from radiolabeled aspartate in a particulate fraction from cat brain extracts, but not in the water soluble fractions. Knizley designated the enzyme activity he observed as acetyl-CoA-L-aspartate *N*-acetyltransferase (ASP-NAT). Goldstein (1969) returned to the study of NAA synthesis a decade after his first report, and in these later studies he concluded that NAA synthesis was associated with the particulate fraction from brain, including microsomal and mitochondrial fractions. He also reported that the synthetic capacity for NAA was substantially higher than reported previously indicating that NAA was not metabolically inert. Research into NAA synthesis did not resume until a number of years later when Truckenmiller et al. (1985) solubilized and characterized the enzyme from brain homogenates and showed that the enzyme acetylated aspartate 10 times more efficiently than it acetylated glutamate. They also found that the enzyme showed a 10-fold variation in activity levels in the nervous system, with the highest activity in brainstem and spinal cord, and the lowest activity in the retina. No enzyme activity could be detected in the pituitary gland, or in heart, liver, and kidney. The enzyme was designated as L-aspartate *N*-acetyltransferase (Asp-NAT or ANAT) and given the enzyme commission (EC) number of EC 2.3.1.17.

After another protracted gap in the research, two laboratories took up the task of purifying Asp-NAT and attempted to determine its subcellular localization. Madhavarao et al. (2003) employed several techniques to purify Asp-NAT activity from brain homogenates, including anion exchange chromatography, native gel electroelution, and size exclusion HPLC. They reported a number of findings on Asp-NAT activity including confirming that the enzyme was highly specific for aspartate, with less than 3% activity directed to the acetylation of glutamate, asparagines, or glutamine, and no detectable capacity to acetylate the dipeptide aspartylglutamate. Looking at enzyme activity relative to the concentration of the two substrates, aspartate and acetyl CoA, they found the Km values to be 580 and 58 μM, respectively. They also noted end-product inhibition of the enzyme by NAA and coenzyme A (IC values of 850 and 420 μM, respectively). Size exclusion chromatography and native gel electrophoresis both indicated that the enzyme was most likely associated with a protein complex that had a molecular weight of ~670 kDa. Finally, subfractionation studies using differential centrifugation showed that enzyme activity was present in myelin, mitochondrial, and synaptosomal fractions. Very similar results were reported by Lu et al. (2004) who used differential centrifugation methods to fractionate brain homogenates into mitochondrial, intermediate, and microsomal fractions and found Asp-NAT activity in all three. Lu et al. found that the microsomal fractions contained the greatest percentage of Asp-NAT activity, although a significant proportion of total activity was lost upon density gradient centrifugation. In addition Lu et al. reported somewhat higher enzyme Km values for aspartate and acetyl CoA (910 and 169 μM, respectively). With regard to the subcellular localization data based on subfractionation methods one caveat should be noted. If neuronal mitochondria are indeed a site of significant Asp-NAT localization then any methods employing brain homogenates will underestimate the mitochondrial component because neuronal mitochondria only comprise a subset of all brain mitochondria and it is difficult to isolate the neuronal mitochondria from synaptic terminals and myelin.

Using radiolabeled precursors to quantify mitochondrial and cytosolic synthetic sites our laboratory employed two metabolic precursors for NAA synthesis that would be utilized differentially in the two sites. We hypothesized that aspartate, which is exported from mitochondria in exchange for glutamate, would be preferentially acetylated in the cytoplasm, whereas malate, which is taken up avidly by mitochondria, would be converted to aspartate (via transamination of oxaloacetate) and acetylated predominantly in the mitochondrial compartment (Arun et al., 2009). In this study employing a cell culture system to model NAA synthesis, as expected, both substrates resulted in the synthesis of radiolabeled NAA. However, the ratio of radiolabeled NAA to radiolabeled aspartate was found to be over 20 times greater when malate was used as substrate, as compared with using labeled aspartate as substrate. This finding was interpreted to indicate that the mitochondrial compartment provided a greater contribution to NAA synthesis than the cytoplasmic compartment. Using the same cell culture system, another research group recently found that the electron transport chain inhibitor antimycin A reduced NAA levels, further substantiating that some proportion of NAA synthesis occurs in mitochondria (Li et al., 2013).

In 2010 the gene for Asp-NAT was finally identified as *Nat8l* which opened up new avenues for characterizing and localizing the enzyme (Ariyannur et al., 2010a; Wiame et al., 2010). Wiame et al. (2010) used transfection of Myc-tagged *Nat8l* in Chinese hamster ovary (CHO) cells and primary neuronal cultures to show that Asp-NAT protein colocalized with endoplasmic reticulum, but not with Golgi apparatus or mitochondria. These investigators also used targeting prediction programs to analyze the *Nat8l* gene sequence and did not find a mitochondrial targeting sequence. Using immunohistochemistry in a neuronal cell line in culture, Ariyannur et al. (2010a) employed affinity purified antibodies to a specific *Nat8l* peptide sequence and found partial colocalization with mitochondria. In a more recent study using transfection of the gene for Asp-NAT into several cell types, Tahay et al. (2012) reported that Asp-NAT expression was restricted to the endoplasmic reticulum, and was not present in mitochondria. Further, they used transfection of truncated forms of the gene to show that Asp-NAT appeared to be targeted to the ER membrane by a hydrophobic loop that connects two regions of the catalytic domain.

### NAA SYNTHESIS AND THE MITOCHONDRIAL MALATE-ASPARTATE SHUTTLE

Mitochondria express a number of solute carrier complexes that move anaplerotic metabolites into the mitochondrial matrix and cataplerotic metabolites out of the matrix. The primary mitochondrial aspartate-glutamate carrier expressed in brain, heart, skeletal muscle, and several other tissues is known as aralar1 (Del Arco et al., 2002), which is part of a larger complex that comprises the so-called mitochondrial malate-aspartate shuttle. The malate-aspartate shuttle functions to move reducing equivalents into the mitochondrial matrix in the form of malate, whereas the major mitochondrial output through this complex is aspartate. Specifically, aralar1 moves cytoplasmic glutamate into mitochondria, while moving mitochondrially synthesized aspartate out. Without aralar1, brain mitochondrial glutamate import and aspartate export are crippled. Studies with aralar1 knockout mice showed a dramatic drop in brain aspartate levels, with a concomitant reduction in NAA synthesis (Jalil et al., 2005; Satrustegui et al., 2007).

The connection between a lack of aralar1 expression and dramatically reduced NAA synthesis has at least two potential explanations. First, as suggested by Jalil et al. (2005) it could be due to the lack of mitochondrial aspartate output, which in turn would limit substrate availability for microsomal Asp-NAT to synthesize NAA. The other possible explanation is that the lack of glutamate uptake into neuronal mitochondria prevents intramitochondrial aspartate synthesis via the aspartate aminotransferase reaction which converts glutamate and oxaloacetate into α-ketoglutarate and aspartate. In this case the lack of intramitochondrial aspartate synthesis would be the limiting factor in NAA synthesis, leading to decreases in NAA levels. It is also possible that both of these mechanisms are responsible for the large drop in NAA levels observed in aralar1-deficient mice. One of the more interesting outcomes of aralar1 deficiency in addition to the large decrease in brain NAA levels is hypomyelination (Jalil et al., 2005; Wibom et al., 2009). The hypomyelination is hypothesized to result from the lack of availability of NAA and this conclusion is supported by the fact that galactocerebrosides, one of the myelin lipid classes that are reduced in Canavan disease (Madhavarao et al., 2005; Arun et al., 2010b), were also reduced in aralar1 knockout mice.

### NAA SYNTHESIS RATES

Burri et al. (1991) were the first to investigate NAA synthesis at specific time points during brain development and showed that Asp-NAT activity in rats increased almost linearly from postnatal day 3 to day 35, and the increase in enzyme activity was paralleled by a corresponding increase in brain NAA levels. Using ^13^C-labeled glucose infusions and ^13^C-MRS of tissue extracts from several brain regions Tyson and Sutherland (1998) found the incorporation rate into labeled NAA was relatively low as compared with the labeling of glutamate, or the neuronal dipeptide *N*-acetylaspartylglutamate (NAAG). In this study the fractional enrichment of NAA after a 200 min ^13^C-glucose infusion was 3%, whereas the fractional enrichment of NAAG was 20% and that of glutamate 25.2%, indicating that in the uninjured adult rat brain the turnover of NAA was relatively slow. In the first use of ^13^C-MRS to study NAA synthesis non-invasively in the human brain, Moreno et al. (2001) estimated that NAA was synthesized in humans at a rate of 9.2 ± 3.9 nmol/min/g. These results were based on measurements from four adults and two children using ^13^C-labeled glucose infusions to investigate ^13^C enrichment in NAA. The calculated NAA synthesis rates from glucose in the four adults were very similar averaging around 8.9 ± 1 nmol/min/g, whereas the calculated rates in the two control children were quite divergent (4.3 and 15.1 nmol/min/g). Compared with the synthesis rates of other brain metabolites the NAA synthesis rate was one to two orders of magnitude lower (e.g., glutamine synthesis rate = 400 nmol/min/g). Moreno et al. also measured NAA synthesis rates in the brains of three children with Canavan disease. NAA concentrations in the brains of Canavan disease patients are elevated, and the synthesis rates are reduced by about 60% relative to controls (3.6 ± 0.1 nmol/min/g).

Overall these results indicate that NAA synthesis in the resting brain is relatively slow when compared with other rapidly synthesized brain metabolites and the total metabolite flux through the TCA cycle. However, the incorporation of carbon from glucose into the aspartate moiety of NAA is an indirect process involving numerous enzymatic steps and dilution with TCA cycle intermediates such as oxaloacetate. It was observed that ^13^C does not appear in NAA until 50 min after ^13^C-glucose infusion was initiated. This is a substantially longer delay than in the case of glutamate or aspartate, in which ^13^C enrichment could be observed as early as 10 min after the start of labeled glucose infusion. Data on the relatively slow but continuous synthesis of NAA in resting adult animals is more suggestive of a metabolic role in biosynthetic reactions (e.g., lipid synthesis and protein acetylation reactions) than in a direct energy derivation role.

Additional studies of NAA synthesis using ^13^C mass-labeled glucose indicated similar synthetic rates for NAA of ~10–12 nmol/min/g (0.6–0.7 μmoles/g/h) with a calculated complete turnover occurring every 2–3 days (Choi and Gruetter, 2004). A later study using similar methods found a lower rate of synthesis in adult rats of only 0.19 μmoles/g/h (Xu et al., 2008) and these authors suggested that the discrepancy between their results and those of Choi and Gruetter may have been due to the localization and size of the voxel used for measurements. The Choi and Gruetter study used a larger voxel that included substantial amounts of white matter, whereas the studies by Xu et al. employed a smaller voxel localized to cerebral gray matter. This would suggest that NAA turnover was significantly higher in white matter. However, it is important to note that all of these studies were done in adult rats, rather than in developing rats when myelination is taking place. It is likely that if these experiments were repeated in developing rats that the observed NAA synthesis rates would be higher, especially in white matter. Another important consideration is that NAA synthesis rates have never been assessed after brain injury. One preliminary investigation examined NAA synthesis rates using ^13^C-MRS with isotope-labeled glucose infusions in Alzheimer patients to determine if the rate of NAA synthesis was regulated in disease states. They found that NAA synthesis was substantially higher in Alzheimer patients, and they hypothesized that this was reflective of a compensatory mechanism where a loss of neurons resulted in higher synthesis rates in the remaining neuronal population (Harris et al., 2006). It will be important for future studies to examine the rate of NAA synthesis from precursors in experimental TBI to determine if, as is the case with Alzheimer disease, the rate is increased significantly during recovery.

### LOCALIZATION OF NAA

The neuronal localization of NAA was inferred in early studies. Benuck and D’Adamo (1968) tested NAA synthesis and hydrolysis in several tissues and found that only the nervous system synthesized NAA, but that many tissues including kidney, liver, heart, and mammary gland catabolized NAA (D’Adamo and Yatsu, 1966). Interestingly, they found that some tissues, for example kidney, avidly took up and metabolized NAA to CO_2_, whereas other tissues took up NAA and converted it to lipids, including brain and mammary gland. They concluded that NAA was only synthesized in the nervous system, but that it could be metabolized in many tissues. Nadler and Cooper (1972a) analyzed various CNS tumors and compared NAA levels to normal brain tissue levels and upon finding that tumors had substantially lower levels of NAA concluded that NAA was predominantly localized in neurons. Chromatographic analyses of different tissue extracts indicated that NAA was present in the brain at concentrations over 100 times greater than that found in non-neural tissues, and this finding further strengthened the case that neurons were the major source of NAA (Miyake et al., 1981). An *in vitro* investigation of various brain cell types using MRS and high performance liquid chromatography indicated that neurons such as cerebellar granule cells contained high levels of NAA, whereas astrocytes and mature oligodendrocytes contained undetectable levels (Urenjak et al., 1992). High NAA levels were also found in oligodendrocyte-type-2 astrocyte (O-2A) progenitor cells and immature oligodendrocytes.

**FIGURE 1:**
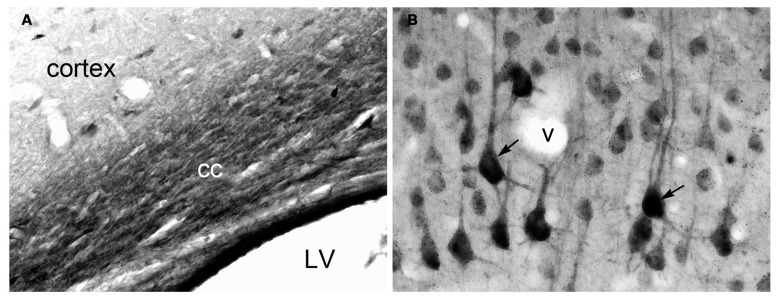
**Immunohistochemistry of NAA in specially fixed rat brain tissue using highly purified polyclonal antibodies to protein-coupled NAA.** NAA immunoreactivity was observed in virtually all fiber pathways, such as the corpus callosum [cc in **(A)**]. In gray matter, NAA immunoreactivity was strong in large cortical pyramidal neurons [arrows in **(B)**], and moderate in smaller cortical neurons **(B)**. NAA was also observed in many neuronal dendrites. Methods described in Moffett and Namboodiri (1995). Images taken with 20× **(A)** and 40× **(B)** objectives. LV, lateral ventricle; v, blood vessel.

Utilizing specific monoclonal (Simmons et al., 1991) and polyclonal (Moffett et al., 1991) antibodies and specialized tissue fixation methods, the neuronal localization of NAA was confirmed via immunohistochemistry. Improvements in those methods allowed for the detailed visualization of NAA in brain tissue sections, and showed that the distribution of NAA in the nervous system did not match the distribution pattern for the related neuronal dipeptide NAAG (Moffett et al., 1993; Moffett and Namboodiri, 1995, 2006). NAA was observed in most neurons, with staining intensities ranging from moderate in hippocampal granule cells to strong in cortical pyramidal neurons. NAA was also observed in the axons of numerous fiber tracts such as the corpus callosum (**Figure 1**).

## NAA, ASPA AND LIPID SYNTHESIS

The first description of the specific enzymatic activity of ASPA was given by Birnbaum et al. (1952). They distinguished aminoacylase I and aminoacylase II derived from kidney based on substrate specificity. Aminoacylase I had a broad specificity for *N*-acetylated amino acids, whereas aminoacylase II (ASPA) was highly specific to NAA. As noted above, Benuck and D’Adamo (1968) found both uptake and utilization of NAA in various non-neural tissues. They showed that radiolabeled NAA was metabolized to both fatty acids and to CO_2_ demonstrating that NAA-derived acetate could be used for either energy production or lipid synthesis, two metabolic pathways tied directly to acetyl CoA utilization. This implied that after NAA is hydrolyzed by ASPA, the resultant acetate is converted to acetyl CoA for further use. Nadler and Cooper (1972b) found that radiolabeled NAA when injected into the brain was metabolized rapidly by deacylation in a relatively small but very active metabolic compartment, and linked the metabolism of NAA to the TCA cycle and glutamate synthesis. D’Adamo et al. (1968) injected NAA that had been radiolabeled on the acetate into the brains of developing rats and found that the label was incorporated mainly into long chain C16 and C18 fatty acids. They proposed that NAA was a mechanism of carbon transport out of mitochondria for fatty acid synthesis. However, at the time it was not known that NAA was present primarily in neurons (Urenjak et al., 1992; Moffett and Namboodiri, 1995), whereas ASPA was present primarily in oligodendrocytes (Baslow et al., 1999; Klugmann et al., 2003; Madhavarao et al., 2004; Moffett et al., 2011), indicating that trans-cellular transport of NAA was involved in some or most of its metabolism in the CNS.

### NAA AND LIPID SYNTHESIS

**FIGURE 2:**
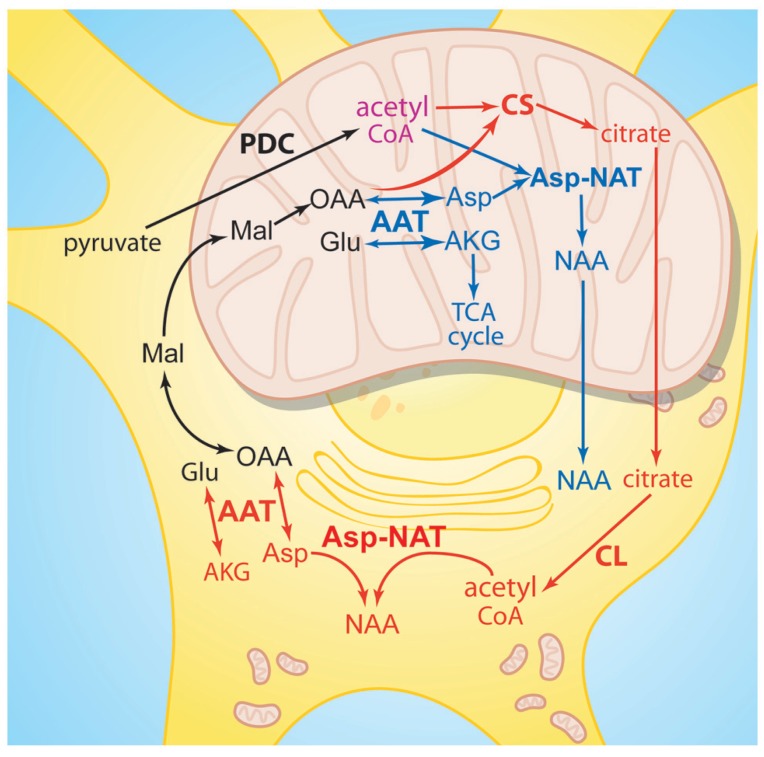
**Similarities between NAA synthesis and citrate synthesis.** Acetyl CoA derived from pyruvate in neuronal mitochondria can be oxidized for ATP production in the TCA cycle, or it can be utilized by two different enzyme pathways to produce metabolites for export to the cytoplasm. Acetyl CoA and oxaloacetate can be converted to citrate via citrate synthase (red pathway), or acetyl CoA and aspartate can be converted to NAA via Asp-NAT (blue pathway). NAA is also synthesized in the endoplasmic reticulum by cytoplasmic Asp-NAT using acetyl CoA derived from citrate. Citrate is then metabolized to acetyl CoA within the cell where it was synthesized, whereas NAA is predominantly transported to other cells such as oligodendrocytes for metabolism. AAT, aspartate aminotransferase; AKG, alpha-ketoglutarate; Asp, aspartate; Asp-NAT, aspartate *N*-acetyltransferase; CL, citrate lyase; CS, citrate synthase; Glu, glutamate; Mal, malate; OAA, oxaloacetate; PDC, pyruvate dehydrogenase complex.

Neurons provide key metabolites to their ensheathing oligodendrocytes for the purposes of myelination, myelin maintenance, and myelin sheath repair, including choline, palmitate, acetate, phosphate, and ethanolamine (Ledeen, 1984). NAA is among the trophic neuronally derived metabolites that are transferred to oligodendrocytes for use in myelination and myelin repair. In order for NAA-derived acetate to participate in lipid synthesis after ASPA-mediated hydrolysis the acetate groups must be activated by conversion to acetyl CoA (D’Adamo and Yatsu, 1966; Burri et al., 1991; Mehta and Namboodiri, 1995). NAA is not a primary source of acetyl CoA as is the case with pyruvate. The synthesis of NAA requires the utilization of existing acetyl CoA and therefore NAA synthesis consumes a portion of brain acetyl CoA stores. Therefore NAA may be acting as a storage and transport form of acetate in the CNS that can be used for subsequent *de novo* synthesis of acetyl CoA, especially in oligodendrocytes (Ariyannur et al., 2010a). Ledeen and coworkers injected radiolabeled NAA into the eye to show that when NAA was transported down the axons the radioactive acetate was incorporated into the myelin lipids in the optic nerve (Chakraborty et al., 2001). This showed that NAA in neurons supplies some of acetyl groups for the synthesis of myelin-associated lipids in oligodendrocytes. NAA supplies ~1/3 of the requisite acetyl CoA for myelin lipid synthesis during brain development and myelination (Wang et al., 2009), with the remaining 2/3 being supplied by citrate produced in oligodendrocyte mitochondria (**Figure 2**). Citrate is produced from acetyl CoA in mitochondria, and the citrate is exported to the cytoplasm where it is reconverted to acetyl CoA by the enzyme citrate lyase (also known as ATP citrate lyase). We have proposed that NAA may be acting as a parallel pathway for supplying additional acetyl CoA to oligodendrocytes (Moffett et al., 2007), which would be more critical during the period of intensive myelination that begins after birth. Unlike citrate, which is converted to acetyl CoA in the same cell, NAA would be transferred to oligodendrocytes where it is reconverted to acetyl CoA by the sequential action of ASPA and acetyl CoA synthase-1 (AceCS1, discussed in more detail below). It is unknown what percentage of acetyl CoA associated with myelin repair and remyelination is derived from NAA after brain injury. Studies using NAA radiolabeled on the acetate moiety could be employed to study this using experimental TBI in rats.

### ASP-NAT EXPRESSION IN BROWN ADIPOSE TISSUE

A recent study revealed that *Nat8l*, the gene encoding the NAA biosynthetic enzyme Asp-NAT, is expressed strongly in brown adipose tissue (BAT; Pessentheiner et al., 2013). To date this is the only tissue other than the CNS where strong *Nat8l* expression has been found. Expression in BAT was similar to expression levels in the brain, whereas expression in white adipose tissue was much lower. Pessentheiner and colleagues found that overexpression of *Nat8l* in BAT cells increased glucose incorporation into neutral lipids, and increased lipolysis suggesting that Asp-NAT expression facilitated lipid turnover in these cells. These investigators also found that when *Nat8l* expression in BAT cells was reduced by RNA silencing, as well as in BAT from *Nat8l* knockout mice, the expression of citrate lyase was enhanced. They proposed that in BAT, as has been suggested for lipid synthesis in oligodendrocytes in the brain (Moffett et al., 2007), the NAA to acetyl CoA pathway may be a parallel pathway for lipogenesis that complements the citrate lyase pathway. However, unlike in the CNS, the NAA produced in brown adipocytes would be utilized in the same cell type, rather than being transferred to a different cell type (e.g., oligodendrocytes) for metabolism.

The use of NAA for synthesizing a portion of myelin lipids during brain development, rather than exclusively using glucose, is unusual because the brain relies on glucose for its metabolic needs more than any other organ (Ramos et al., 2011). Adult mammals utilize glucose as the primary energy deriving metabolite and carbon source in the brain, but it is well documented that ketone bodies are preferred over glucose as an energy source for neurons and oligodendrocytes during brain development (Edmond et al., 1987). It has also been shown that acetate is released from the liver along with ketone bodies to provide another carbon source to various tissues of the body, including the brain (Ballard, 1972; Yamashita et al., 2001). Using radiolabeled metabolites Edmond et al. (1987) showed that ketone bodies were nine times more effective than glucose for supporting oligodendrocyte respiration during brain development and myelination. Postnatal myelination occurs when infant mammals are suckling, and glucose availability in the diet is low. Therefore, ketone bodies and acetate are critical energy metabolites for oligodendrocytes during brain development, in contrast with adults where glucose is the primary energy and carbon source.

Traumatic brain injury results in disrupted lipid metabolism, oxidative damage to, and degradation of mitochondrial phospholipids (Adibhatla et al., 2006; Singh et al., 2006), damage to subcortical white matter and delayed axonal degeneration throughout the brain (Hall et al., 2005; Park et al., 2008). Because NAA-derived acetate is one of the building blocks for myelin lipid synthesis in the brain, and that NAA levels are substantially reduced after TBI, we have pursued the concept that a potent source of acetate which can cross the blood brain barrier would be useful for the treatment of brain injuries. Glyceryl triacetate (GTA), the triester of acetate and glycerol, fulfills these criteria. We have previously shown that orally administered GTA can substantially increase brain acetate levels (Mathew et al., 2005), and can be used to increase myelin lipid synthesis in neurological disorders that involve hypomyelination or demyelination (Arun et al., 2010b). A logical extension of this finding is that GTA may be useful in other brain disorders that involve remyelination, including TBI.

## NAA, ACETATE, AND ENERGY METABOLISM

Traumatic brain injury results in the disruption of brain energy metabolism and the resultant energy deficit is proportional to the degree of damage (Marklund et al., 2006). The levels of NAA and ATP are reduced immediately after brain injury and remain depressed for hours, days or weeks depending on injury severity (Signoretti et al., 2001; Tavazzi et al., 2005; Vagnozzi et al., 2005; Arun et al., 2010a). These reductions are indicative of metabolic impairment and the depletion of energy stores in the brain, and it is very likely that the reduction in NAA levels is tied to the loss of the immediate precursor, acetyl CoA, after brain injury (Vagnozzi et al., 2007).

### NAA AND MITOCHONDRIAL FUNCTION

Patel and Clark (1979) provided the first clear experimental evidence that NAA synthesis was associated with mitochondrial energetics and mitochondrial carbon transport in the brain. Subsequently, a number of investigators have provided multiple lines of evidence supporting the association between NAA and neuroenergetics. More recently, Signoretti and associates have provided extensive preclinical and clinical data in support of this connection in the context of TBI. However, the precise relationship between NAA metabolism and energy derivation in the nervous system remains largely unclear.

We have proposed a multi-functional model of NAA metabolism involving its synthesis in neuronal mitochondria and endoplasmic reticulum (Madhavarao et al., 2003; Madhavarao et al., 2005; Arun et al., 2009). The key aspect of the model is the connection between NAA metabolism and acetyl CoA generation and utilization in the nervous system (Ariyannur et al., 2010a). Because NAA synthesis requires the use of existing acetyl CoA stores, NAA synthesis can only occur when acetyl CoA levels are in excess of current neuronal metabolic requirements. This means that when energy reserves are low as in the case of brain injury, NAA synthesis will be compromised. NAA may be acting as an acetyl CoA precursor or buffer to help regenerate some acetyl CoA during the initial response to brain injury. This is consistent with data from brain injury studies showing that NAA, ATP, and acetyl CoA are all reduced rapidly after brain injury and that their levels return in concert with recovery (Vagnozzi et al., 2007).

**FIGURE 3:**
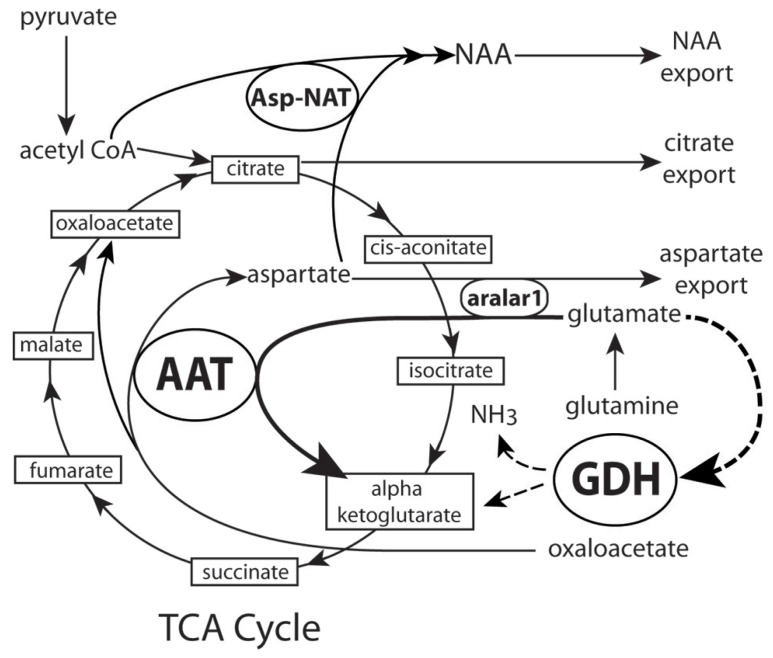
**Proposed schematic of NAA synthesis in neuronal mitochondria.** Dashed lines show α-ketoglutarate pathway through GDH that is bypassed by synthesis through AAT. As more pyruvate enters the cycle NAA synthesis is increased, aspartate export from mitochondria is decreased and NAA export is increased. See text for details. AAT, aspartate amino-transferase (also known as glutamate oxaloacetate transaminase); Asp-NAT, aspartate *N*-acetyltransferase; GDH, glutamate dehydrogenase; NH_3_, ammonia.

We have proposed that another mechanism in which NAA synthesis serves a neuroenergetic role in mitochondria by facilitating the oxidation of glutamate as an additional energy source (Madhavarao et al., 2003; Madhavarao and Namboodiri, 2006; Moffett et al., 2007). The synthesis of NAA in mitochondria requires continuous production of aspartate from oxaloacetate. This is accomplished by the action of the enzyme aspartate aminotransferase (also known as glutamate oxaloacetate transaminase (**Figure 3**). Aspartate aminotransferase converts oxaloacetate and glutamate to aspartate and α-ketoglutarate, and the aspartate can then be converted to NAA, while the α-ketoglutarate can enter the TCA cycle for energy derivation. This enzymatic reaction increases α-ketoglutarate formation from glutamate and energy production via the citric acid cycle. In this way, the demand for ATP in neurons can be augmented by oxidation of glutamate via the aspartate aminotransferase pathway. Glutamate dehydrogenase is another enzyme that converts glutamate to α-ketoglutarate. Glutamate dehydrogenase is expressed in the mitochondria and endoplasmic reticulum of neurons and glia, and it catalyzes the deamination of glutamate to alpha-ketoglutarate and ammonia using either NAD or NADP as cofactors (Mastorodemos et al., 2005, 2009). Once glutamate is imported into mitochondria it is predominantly destined for conversion to α-ketoglutarate. By preferentially using the aspartate aminotransferase reaction instead of the glutamate dehydrogenase reaction to generate α-ketoglutarate, neuronal mitochondria would circumvent the generation of ammonia associated with the glutamate dehydrogenase reaction. The aspartate aminotransferase reaction also generates additional aspartate from oxaloacetate, and the aspartate can then be acetylated by Asp-NAT to form NAA. A recent study has shown that the decrease in ATP in blast mediated TBI is associated with a decrease in aspartate aminotransferase expression in the brain (Arun et al., 2013). Reduced aspartate aminotransferase activity in neuronal mitochondria would limit both NAA synthesis from aspartate, and α-ketoglutarate synthesis from glutamate, thus reducing the brain’s capacity to generate energy.

A number of other findings in the literature are consistent with or supportive of these mechanisms. First, inhibition of aspartate aminotransferase activity by β-methylene-aspartate was shown to decrease oxygen consumption in the presence of glutamate and malate as energy sources (Cheeseman and Clark, 1988). Second, efflux of NAA was increased, whereas that of aspartate was decreased with increasing concentrations of pyruvate in the presence of glutamate and malate as substrates in mitochondria isolated from rat brain (Patel and Clark, 1979). Third, ATP synthesis, NAA synthesis, and oxygen consumption were all reduced similarly in isolated brain mitochondrial preparations after application of several respiratory chain inhibitors (Bates et al., 1996). There are numerous experimental paradigms that have demonstrated decreases in NAA levels when brain energy metabolism is impaired by different mechanisms. Another experimental paradigm involved rat and primate models of progressive striatal neurodegeneration induced by the mitochondrial toxin 3-nitropropionic acid (3-NP). In rats treated with 3NP, NAA decrease was observed selectively in the striatum before any cell loss and was associated with motor dysfunction. Also, a similar selective and early striatal decrease in NAA was observed in 3-NP treated primates (Dautry et al., 2000). The initial decrease in striatal NAA was partially reversed after 3 weeks of 3-NP withdrawal. The mouse model for Huntington disease is another experimental paradigm wherein decreases in NAA have been documented. The drop in brain NAA levels commenced at about 6 weeks of age and coincided with the onset of symptoms (Andreassen et al., 2001). These decreases in NAA occurred in the absence of any neuronal cell death. Also, dietary creatine supplementation significantly improved survival, slowed development of brain atrophy and delayed decreases in NAA (Andreassen et al., 2001). These results also indicate that the levels of NAA in the brain reflect the health of neurons and their mitochondria, and can therefore act as reliable markers for monitoring neuronal energy impairment and dysfunction.

Using combined proton MRS for NAA levels and ^31^P MRS for ATP and ADP levels, Pan and Takahashi (2005) showed that NAA levels in the uninjured human brain (occipital lobe and hippocampus) correlate with ADP rather than ATP. These investigators speculated that this ties NAA synthesis positively to ADP generation, perhaps indicating that NAA synthesis was responsive to a decreasing energy charge in brain.

Recent studies with aralar1 knockout mice also provide support for a bioenergetic role for NAA. Aralar1 knockout mice show reduced neuronal respiration on glutamate plus malate (Jalil et al., 2005). It is possible that the acetylation of aspartate by asp-NAT in neuronal mitochondria could combine with the action of aralar1 to remove product inhibition of the aspartate aminotransferase reaction, and this would facilitate the conversion of glutamate to α-ketoglutarate, which can then enter the tricarboxylic acid (TCA) cycle for energy production (see **Figures 2 and 3**). Based on the above model it is reasonable to state that formation of NAA facilitates glutamate oxidation, but there is no evidence to indicate that NAA *per se* participates directly in any step of mitochondrial energy production.

As noted above, *Nat8l* was recently found to be expressed strongly in BAT, and was associated with increased lipid turnover. In the same study *Nat8l* was shown to be present predominantly in mitochondria in brown fat cells, as opposed to endoplasmic reticulum (Pessentheiner et al., 2013). Further, it was shown that *Nat8l* overexpression increased the number of mitochondria per cell, and increased oxygen consumption rate. *Nat8l* overexpression also dramatically upregulated the expression of BAT marker genes including UCP1 (uncoupling protein 1). UCP1 acts to uncouple oxidative phosphorylation from ATP synthesis by reducing the proton gradient across the inner mitochondrial membrane, thus generating heat. Pessentheiner et al. further demonstrated that overexpression of an *Nat8l* mutant gene that produces a protein with no NAA biosynthetic capacity into BAT cells did not increase BAT marker gene expression indicating that Asp-NAT enzymatic activity is required for increased BAT gene expression. These findings link Asp-NAT not only to mitochondrial function, but also to gene expression (see Nuclear Protein Acetylation below).

### NAA AND ATP LOSS IN TBI

*N*-Acetylaspartate levels are substantially reduced after severe brain injury or stroke, and levels may recover after days or weeks depending on the severity of the injury (Wardlaw et al., 1998; Signoretti et al., 2010, 2011; Vagnozzi et al., 2010). Recovery of NAA to normal levels is typically seen after milder brain injuries, but not in the case of severe injuries, or in the case of multiple milder injuries spaced closely in time as often occurs in contact sports. Studies employing experimental TBI in rats strongly link NAA to neuronal energetics. Signoretti et al. (2001) used a sensitive HPLC method to show that moderate experimental TBI led to maximal reductions in NAA at 15 h post-injury (-46%) and maximal ATP reductions at 6 h post-injury (-57%). The levels of both metabolites recovered to near normal levels by 5 days after injury indicating that moderate TBI results in a severe energy impairment that does not involve extensive neuronal death. In the same study severe TBI was found to result in even greater energy depletion with NAA and ATP levels falling by ~60% by day five after injury, with no indication of recovery of normal levels for either compound suggesting extensive and permanent loss of neurons. Levels of ATP and NAA were depleted even further when severe TBI was coupled with temporary hypoxia-hypotension (90 and 80%, respectively 48 h after injury).

Subsequent studies defined a window of increased vulnerability to a second injury event whereby a single moderate TBI resolved after ~5 days, with recovery of NAA and ATP levels (Vagnozzi et al., 2007; Signoretti et al., 2011). However, when a second moderate TBI event occurred 3 days after the initial injury, then NAA and ATP levels did not recover, and outcomes were substantially poorer. It was also found that if the second moderate injury event occurred five or more days after the first event, when NAA and ATP levels had already recovered, then the second event also was followed by recovery of brain energy stores. These findings indicate that the energy depletion associated with moderate brain injury leaves the brain much more vulnerable to a second injury event.

### NON-GLUCOSE ENERGY SOURCES IN THE TREATMENT OF TBI

The brain derives most of its energy from glucose, but the brain can also make use of alternate energy sources including ketone bodies, glutamate/glutamine, lactate, and acetate. Studies on the response of neurons to hypoglycemia show that neurons can make use of a number of alternative substrates for energy derivation when glucose levels are insufficient (Amaral, 2013). TBI results in an energy deficit in cerebral tissue (Vespa et al., 2005) with a concomitant increase in the uptake and utilization of ketone bodies (White and Venkatesh, 2011). Attempts to compensate for the energy deficit by providing nutrition in the form of glucose or other carbohydrates to TBI and stroke patients can result in hyperglycemia and exacerbate secondary damage (Ginsberg et al., 1980; Welsh et al., 1980). This has led some researchers to explore the use of non-carbohydrate sources of nutrition to improve recovery from stroke and TBI. Robertson et al. (1992) used several non-carbohydrate energy sources, as well as 24 h fasting, in an experimental model of ischemia in rats and compared infarct volume between groups. They administered 1,3-butanediol, triacetin (GTA), tributyrin, and long- and medium-chain triglycerides, as well as a combination of triacetin and tributyrin to rats subjected to temporary middle cerebral artery occlusion. The fasted group had the smallest infarct volumes, and the 1,3 butanediol group had the next smallest infarct volumes. The triacetin/tributyrin group also showed reduced infarct volumes relative to the control group that was given a diet of normal rat chow. These findings indicate that diets which mimic fasting (ketogenic) conditions provide a more suitable post-injury nutritional support for CNS repair.

Lactate has been studied as an alternative nutritive energy source after experimental TBI in rats (Rice et al., 2002). Using the fluid percussion model of TBI Rice and coworkers found that 100 mM lactate infusion 30 min after injury resulted in significantly increased ATP levels in the ipsilateral hemisphere at 3 h post injury. Morris water maze performance on days 11 through 15 after injury were also significantly improved with 100 mM lactate treatment. A more recent study found no improvement in brain energy status with post-injury lactate infusion in rats subjected to severe TBI and the authors attributed this to the reductions in NAD^+^ which then prevented significant pyruvate synthesis from lactate (Prieto et al., 2011). Prins et al. (2004) used 3 h of β-hydroxybutyrate infusion beginning immediately after injury and found that the injury resulted in an 8.5-fold increase in uptake of the ketone body into brain, and that CO_2_ derived from radiolabeled β-hydroxybutyrate increased by nearly 11%. Treatment also reversed the 20% drop in ipsilateral ATP levels observed after injury in the saline treated group. Prins (2008) summarizes the findings by stating: “Whether ketosis is achieved by starvation or administration of a ketogenic diet, the common underlying conditions of low plasma glucose in the presence of an alternative substrate (ketones) have consistently shown neuroprotective effects after various types of brain injuries.”

Acetylcarnitine has been shown to reduce neurological deficits after global cerebral ischemia and reperfusion (Rosenthal et al., 1992) and has also been found to reduce lesion size and improve neurological outcomes in young rats subjected to controlled cortical impact injury (Scafidi et al., 2010a). Scafidi et al. concluded that acetylcarnitine improved cerebral energy metabolism and therefore reducing necrotic cell death due to metabolic failure. This conclusion is supported by studies of experimental spinal cord injury in rats where treatment with acetylcarnitine significantly improved acute mitochondrial function and long term motor function recovery (Patel et al., 2012). Scafidi et al. (2010b) used ^13^C-MRS to show that when acetylcarnitine that was mass labeled on the acetyl moiety was injected into 21- to 22-day-old rats the acetate group was utilized for energy derivation in the TCA cycle, and also became incorporated into a number of brain metabolites. The labeled metabolites that were detected in the mass spectra 2 h after administration included glutamate, glutamine, taurine, creatine, lactate, NAA, and GABA. This labeling pattern indicates metabolism of the acetyl group of acetylcarnitine through the TCA cycle in both neurons and glial cells.

Our laboratory has been investigating the use of GTA as a method of delivering high doses of acetate to the brain. GTA is a hydrophobic molecule that crosses the blood brain barrier and enters cells in the brain without the need of transporters. This high degree of bioavailability may be an important factor in the treatment of brain injury due to compromised cerebral circulation, which can limit the distribution of water soluble compounds. In neuronal and glial cytoplasm GTA is rapidly broken down by non-specific esterases and lipases to generate free acetate and glycerol. Using the unilateral controlled cortical impact model of TBI in rats we found that GTA administration significantly improved NAA and ATP levels in the injured hemisphere 4 and 6 days after injury (Arun et al., 2010a). Therefore, the reduction in brain energy stores resulting from brain injury can be partly reversed by the administration of GTA as a non-glucose energy source. Improvements in motor performance on a Rotarod balance test were also significant 3 days after injury, suggesting that GTA administration may accelerate post-injury recovery rates. The partial restoration of NAA we observed in the injured hemisphere after GTA treatment contrasts with the earlier finding that high doses of GTA given over time to uninjured mice did not increase the normal levels of NAA in the brain (Mathew et al., 2005). The beneficial effects of GTA are likely to be a due to the significant drop in brain acetyl coenzyme A levels associated with TBI (Vagnozzi et al., 2007), which would affect both ATP and NAA synthesis rates. Increasing acetate availability after TBI could partly compensate for the reduced availability of acetyl CoA and increase both ATP and NAA synthesis. GTA has also been found to reduce neuroinflammation (Reisenauer et al., 2011; Brissette et al., 2012; Soliman et al., 2012), which may increase its effectiveness after TBI.

Acetate is not technically a ketone body because it is metabolized by different enzymes than acetoacetate or β-hydroxybutyrate. However, like ketone bodies it can be used for energy derivation and for the synthesis of fatty acids. Therefore, by providing free acetate, GTA may be working in a fashion analogous to ketone bodies, but without the same restriction to young animals and the developing brain. Acetate can be converted to acetyl coenzyme A by the action of acetyl coenzyme A synthetase (Nikolau et al., 2000). Two forms of acetyl coenzyme A synthetase exist in cells, a mitochondrial form (AceCS2) involved in energy metabolism (Fujino et al., 2001), and a nuclear-cytosolic form (AceCS1) involved in lipid synthesis and protein acetylation reactions (Luong et al., 2000; Wellen et al., 2009; Ariyannur et al., 2010b). Therefore, the acetyl CoA produced from GTA can go on to be oxidized in the TCA cycle for energy production in mitochondria (AceCS2), or can enter lipid synthetic pathways for the production of membrane and myelin lipids in the cytoplasm (AceCS1).

### AceCS1 AND AceCS2 LOCALIZATION IN THE BRAIN

**FIGURE 4:**
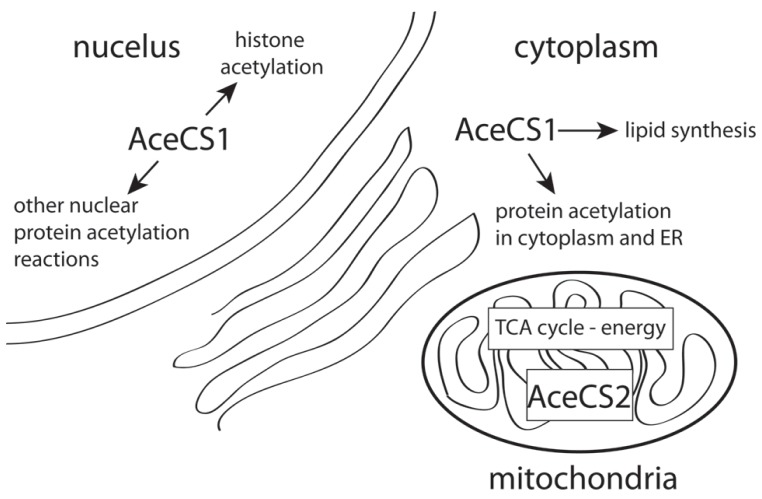
**AceCS1 and AceCS2 have different subcellular distributions.** AceCS1 is localized in the cell cytoplasm where it generates acetyl CoA for lipid synthesis and cytoplasmic protein acetylation reactions. AceCS1 is also localized in cell nuclei where it generates acetyl CoA for histone and other protein acetylation reactions. AceCS2 is localized in mitochondria where it supplies acetyl CoA for energy derivation via the TCA cycle.

**FIGURE 5:**
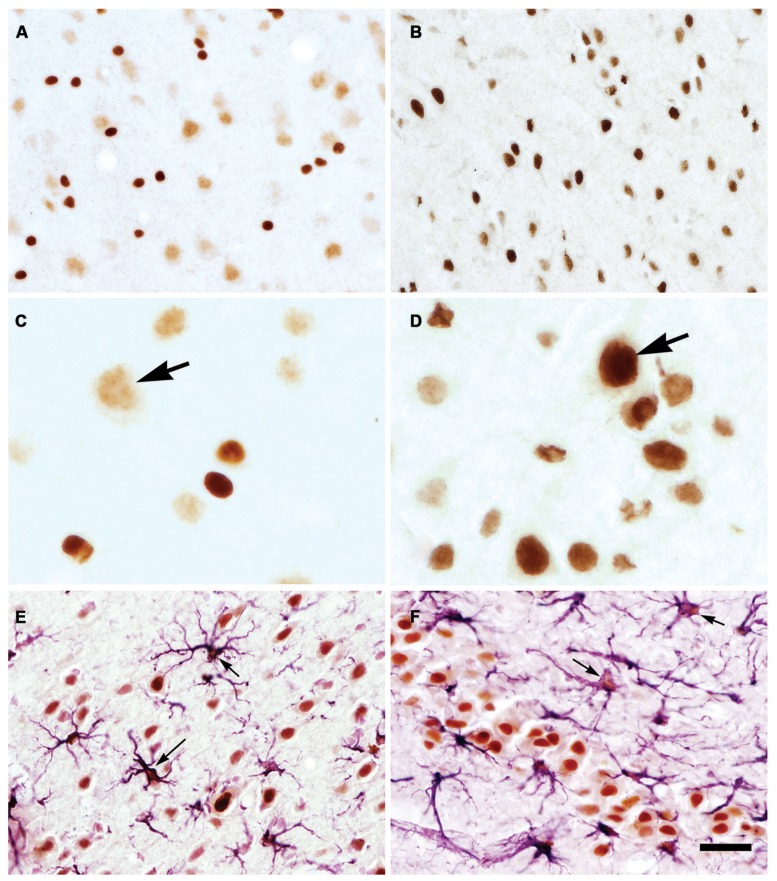
**AceCS1 expression is increased after TBI.** AceCS1 immunoreactivity in control **(A,C)** and 3 days after controlled cortical impact injury on the side ipsilateral to the injury **(B,D–F)**. Lower magnification images of layer V of neocortex are shown in **(A,B)** indicating the increased expression of AceCS1 after injury, with higher magnification of layer V shown in **(C,D)**. Arrows in **(C,D)** show the substantial increase in AceCS1 expression in the nuclei of larger cortical pyramidal cells. **(E)** (layer V neocortex) and **(F)** (CA3 region of hippocampus) show double staining for AceCS1 (orange) and GFAP in astrocytes (purple). A large increase in expression was observed for both AceCS1 and GFAP 3 days after injury. Arrows in **(E,F)** show astrocytes with AceCS1 positive nuclei. Bar = 30 μm **(A,B,E,F)**, 10 μm **(C,D)**. Methods given in Ariyannur et al. (2010b).

A primary factor required for NAA-derived acetate to be used for energy derivation, lipid synthesis, or protein acetylation reactions is the expression of the acetyl CoA synthetase enzymes AceCS1 and AceCS2 (**Figure 4**). AceCS1 had long been thought to be a cytoplasmic enzyme that was primarily involved in acetyl CoA production for fatty acid synthesis (Woodnutt and Parker, 1978; Imesch and Rous, 1984). However, more recent studies have indicated that AceCS1 is a nuclear-cytoplasmic enzyme that is also involved in protein acetylation reactions including histone acetylation (Takahashi et al., 2006; Wellen et al., 2009). Supporting this observation, we have used antibodies to AceCS1 and showed that in the adult rat brain the predominant localization is nuclear, rather than cytoplasmic, and that expression of AceCS1 was substantially upregulated after experimental TBI in rats (Ariyannur et al., 2010b). The greatest increase in AceCS1 expression after TBI was in the nuclei of cells throughout the brain, including both neural and glial cells, but some increased cytoplasmic staining was also observed (**Figure 5**). Previously it has been shown that mRNA levels for the NAA-degrading enzyme ASPA are upregulated as much as fourfold after experimental TBI in rats (Vagnozzi et al., 2007). These findings suggest that the capacity to generate and utilize NAA-derived acetate for lipid synthesis and protein acetylation reactions is substantially increased in response to brain injury (see Nuclear Protein Acetylation and Cytoplasmic Protein Acetylation below).

**FIGURE 6:**
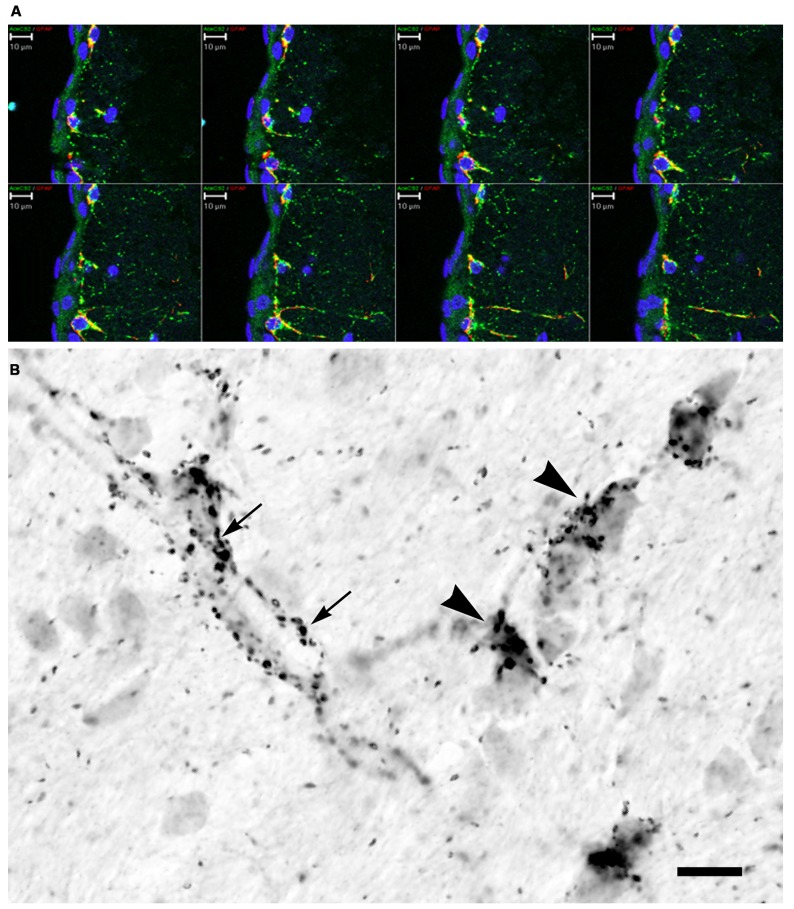
**AceCS2 immunoreactivity in the rat brain using an antibody generated against a 14 AA sequence from the C-terminus of murine AceCS2 (1:5000 dilution).** Immunoreactivity in the adult rat brain was only observed in small punctuate structures, especially in white matter tracts, on some blood vessels and in the pia matter. **(A)** Confocal *z*-series images of immunofluorescence for AceCS2 (green) and the astrocyte marker GFAP (red) at the cortical surface including the pia matter (blue indicates DAPI staining of cell nuclei). Colocalization of AceCS2 and GFAP appears as yellow. This series of images show the merged images at various depths within the tissue slice. AceCS2 was associated with punctuate structures in astrocytes. **(B)** High magnification image of immunohistochemistry in adult rat corpus callosum. The punctuate structures were often closely apposed to blood vessels (arrows) or oligodendrocyte cell bodies (arrowheads). The stained structures ranged in size from ~½ to 1 micron. Bars in **(A,B)** = 10 μm.

**FIGURE 7:**
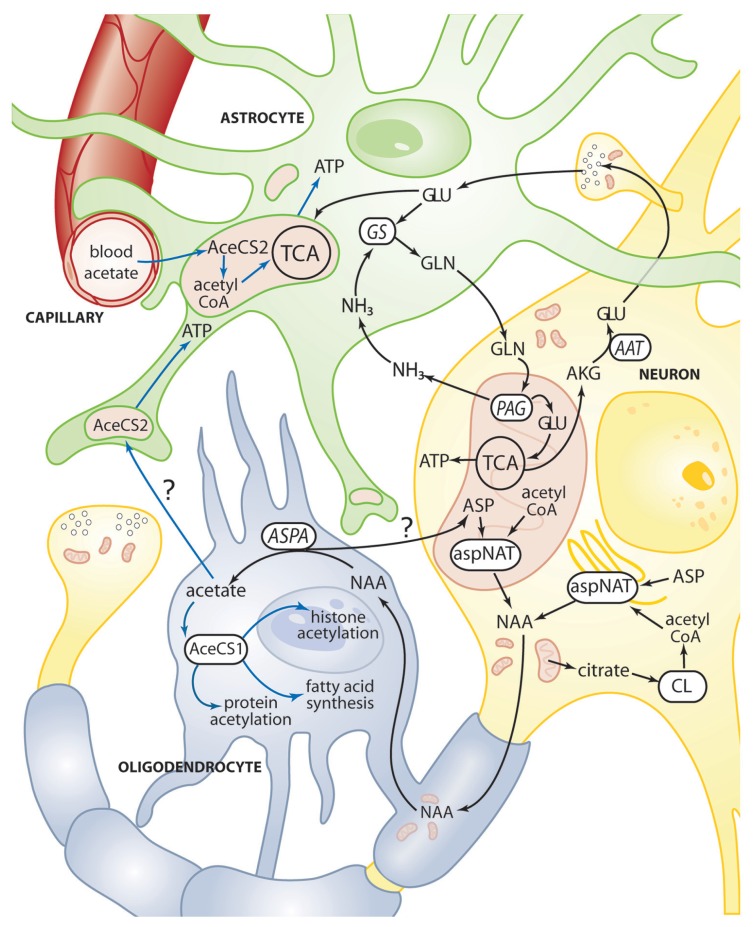
**Schematic diagram depicting some of the metabolic trafficking between neurons, oligodendrocytes, and astrocytes.** A major intercellular cycle between neurons and astrocytes is exemplified by the glutamate-glutamine cycle. A percentage of glutamate is not recycled however, and instead is oxidized for energy derivation in the TCA cycle of neurons and astrocytes. A major metabolite cycle also exists between neurons and oligodendrocytes via NAA that is transferred from axons to oligodendrocytes at axo-glial contact zones between the innermost oligodendrocyte plasma membrane in a myelin segment and the axonal membrane. ASPA in the oligodendrocytes generates free acetate which can then be converted to acetyl CoA by AceCS1, which can then go on to be utilized for fatty acid synthesis, cytoplasmic protein acetylation reactions, and nuclear histone acetylation (blue lines). It is possible that some acetate derived from NAA in oligodendrocytes is transferred to astrocytes for energy derivation though the enzyme AceCS2 present in mitochondria in astrocyte end feet and cellular contact zones. AceCS2 in astrocytes is also in a position to capture blood borne acetate for energy derivation. See **Figures 2** and **3** for details of the reactions in neuronal mitochondria. It is also possible that some NAA-derived aspartate in oligodendrocytes is recycled back to neurons. AAT, aspartate aminotransferase; AceCS1, acetyl CoA synthase-1; AceCS2, acetyl CoA synthase-2; AKG, α-ketoglutarate; ASP, aspartate; ASPA, aspartoacylase; aspNAT, aspartate *N*-acetyltransferase; CL, ATP citrate lyase; GLN, glutamine; GLU, glutamate; GS, glutamine synthase; NH_3_, ammonia; PAG, phosphate activated glutaminase.

AceCS2 is a mitochondrial enzyme that converts free acetate into acetyl CoA for energy derivation through the TCA cycle, and is expressed most strongly in cardiac and skeletal muscle and BAT (Fujino et al., 2001; Sakakibara et al., 2009). AceCS2 is also expressed at relatively lower levels in the brain. We used two different antibodies to localize AceCS2 in the brain and using dual labeling found it to be associated primarily with GFAP-positive astrocytes (**Figure 6A**). In agreement with studies on the tissue distribution of AceCS2 (Fujino et al., 2001) AceCS2 expression was relatively low in the brain. Strong AceCS2 immunoreactivity was observed in punctuate structures that could represent mitochondria in astrocyte end feet on blood vessels and on the surface of other cells including oligodendrocytes (**Figure 6B**). AceCS2 was also associated with similar punctuate structures in the pia matter. It is possible that these apparent intercellular contacts between astrocytes and endothelial cells and oligodendrocytes indicate that astrocytes are making use of acetate from the blood stream, as well as NAA-derived acetate from oligodendrocytes as an energy source (see **Figure 7** below).

### NAA AND METABOLIC INTERACTIONS BETWEEN NEURONS AND GLIA

The classical view of brain energetics focused on glucose as the primary and almost exclusive energy source for the brain. However, the emerging view of energy utilization in the brain is that other metabolites are used by the brain as significant energy sources including glutamate and acetate, which are utilized extensively by astrocytes during normal brain function and in response to pathology. Under resting conditions the metabolic rate of acetate utilization corresponds to between 15 and 25% of the glucose utilization rate (Dienel and Cruz, 2006). When acetate is utilized as a substrate for cerebral metabolism less citrate is used (reduced citrate lyase activity) thus sparing citrate for other uses (Rae et al., 2012). Astrocytes provide the major interface between circulating nutrients and the other cell types in the brain including neurons and oligodendrocytes. As a primary part of the blood brain barrier astrocytes act as a selective filter and nutrient distribution system to the other cell types that do not have such direct access to circulating nutrients. Astrocytes are also the principle users of acetate reaching the brain via the circulation due to their robust uptake capacity, and they oxidize much of the acetate for energy derivation via the TCA cycle (Waniewski and Martin, 1998). However, NAA-derived acetate as well as acetate from other intracellular sources (acetyl CoA, acetylcarnitine, acetylated proteins, etc.) may be utilized locally in the cells where it is generated if an acetyl CoA synthase enzyme is present. For example, some larger neurons in the brainstem and spinal cord of rats express both ASPA (Moffett et al., 2011) and AceCS1 (Ariyannur et al., 2010b) and therefore may be capable of not only NAA synthesis, but also NAA catabolism and regeneration of acetyl CoA from the liberated acetate.

The exceptionally high level of NAA in the nervous system, coupled with the fact that NAA diverts some acetyl CoA from other potential uses, suggests that NAA serves as an acetyl group (carbon) storage molecule that is synthesized when glucose is in excess of minimal system needs. The stored acetate can be reclaimed by the action of ASPA, followed by the action of AceCS1 or AceCS2 to regenerate acetyl CoA. Because some proportion of NAA is transferred to oligodendrocytes for metabolism (Chakraborty et al., 2001) it is part of the metabolic trafficking system between cell types in the nervous system. Studies on the comparative utilization of acetate and glucose revealed a close coupling of the TCA cycle in astrocytes and neurons, and suggest highly cooperative energy derivation in the two cell types (Serres et al., 2008). This coupling may be increased after brain injury. Bartnik-Olson et al. (2010) used the fluid percussion model of TBI to show that oxidation of glucose is reduced in neurons after injury, but that acetate utilization in astrocytes is increased indicating that astrocytes make use of an alternate energy source to maintain brain energy levels in response to injury. Astrocytes increased their incorporation of acetate into glutamine in a time dependent manner during the hypometabolic period after injury, and the trafficking of metabolites between astrocytes and neurons was maintained during the recovery period.

Astrocytes have received the most attention with regard to metabolic trafficking between cell types in the brain, but recently some authors have stressed the importance of oligodendrocytes in intercellular metabolic interactions. This has led to the concept of neuron-oligodendrocyte-astrocyte interactions wherein metabolites given off by one cell type are taken up and utilized by other cell types (Amaral et al., 2013). NAA is one of the key metabolites transferred from neurons to oligodendrocytes to support various metabolic demands for acetyl CoA (**Figure 7**). The aspartate derived from NAA can be utilized for protein synthesis or converted to oxaloacetate by transamination for further metabolism. It is also possible that some of the aspartate is recycled back to neurons.

## NAA AND PROTEIN ACETYLATION – NAA AS AN ACETYL GROUP RESERVOIR

Early indications that NAA was involved in acetylation reactions were provided by Buniatian et al. (1965) who reported that acetylcholine and acetylglucosamine could be synthesized from NAA-derived acetate. They also found that when rat brain cortical slices were incubated in the presence of added glucose, NAA was released to the medium. When glucose was omitted, NAA levels were reduced and aspartate levels in the media increased. This is consistent with the fact that acetyl CoA is required for NAA synthesis. Pyruvate is the end product of glycolysis, so as glucose levels increase so do the levels of pyruvate, which is the immediate precursor for acetyl CoA synthesis in mitochondria (see **Figures 2 and 3**).

### NUCLEAR PROTEIN ACETYLATION

AceCS1 functions to utilize free acetate, and convert it into metabolizable acetyl CoA. Free acetate originates from several sources including the activity of gut bacteria, and the enzyme acetyl CoA hydrolase, which breaks down existing acetyl CoA to release the acetate. This happens in the liver under ketogenic conditions and the liberated acetate is released to the circulation along with ketone bodies (Grigat et al., 1979; Woodnutt and Parker, 1986). The acetate can be taken up from the circulation by other tissues and reconverted to acetyl CoA via AceCS1. AceCS1 was originally thought to be a lipogenic enzyme involved in the utilization of acetate primarily for lipid synthesis (Ikeda et al., 2001). However, another recently discovered function of AceCS1, the nuclear-cytosolic form of the enzyme (Ariyannur et al., 2010b), is to provide acetyl CoA for nuclear histone acetyltransferase enzymes involved in epigenetic gene regulation (Takahashi et al., 2006). In order for transcription-associated enzymes such as RNA polymerase to have access to the appropriate DNA strands and for gene transcription to proceed histone acetylation is required to dissociate histones from DNA, and this is accomplished by the action of histone acetyltransferases (HATs) that act to acetylate lysine residues on histone proteins. Histone deacetylase enzymes (HDACs) act to deacetylate histones and facilitate their binding to DNA thus silencing gene transcription. Inhibiting HDACs prolongs the dissociation of histone proteins from DNA, permitting enhanced gene transcription. Dash et al. (2009) have shown that the HDAC inhibitor sodium butyrate improves learning and memory in rats following experimental TBI. Inhibiting histone deacetylation may enhance gene transcription for cellular repair mechanisms and neuronal plasticity. The drop in acetyl CoA levels after TBI could limit the ability of cells to maintain proper transcription levels due to reduced histone acetylation. We have found that AceCS1 is strongly upregulated in neuronal and glial cell nuclei after TBI (Ariyannur et al., 2010b), suggesting increased enzymatic capacity to synthesize acetyl CoA from free acetate in response to brain injury. We further hypothesized that the strong expression of AceCS1 in cell nuclei provides a mechanism to recycle free acetate generated by the action of HDACs. Reclaiming this acetate would help maintain nuclear acetyl CoA levels especially during times of increased gene transcription, such as in response to injury. ASPA (Hershfield et al., 2006; Moffett et al., 2011) and AceCS1 (Ariyannur et al., 2010b) are localized extensively in the nuclei of oligodendrocytes, and therefore are in a position to provide additional acetyl CoA for nuclear HAT reactions. It is reasonable to hypothesize that this arrangement provides a means for targeting metabolism of NAA-derived acetate to the cell nucleus of oligodendrocytes for intra-nuclear synthesis of acetyl CoA to support acetyltransferase reactions. Such an arrangement would be more robust than relying solely on diffusion of cytoplasmic acetyl CoA into the nucleus, especially at times of increased transcription associated with brain injury.

### CYTOPLASMIC PROTEIN ACETYLATION

One additional deficit associated with reduced acetyl CoA availability after TBI involves the posttranslational acetylation of proteins outside of the nucleus in the endoplasmic reticulum and organelles such as mitochondria. Many cell types that have active protein secretory pathways through the endoplasmic reticulum, such as neurons and oligodendrocytes, are sensitive to disorders of protein mis-folding. Evidence indicates that acetylation and deacetylation of nascent polypeptide chains in the endoplasmic reticulum is required for stabilization and correct folding (Costantini et al., 2007; Spange et al., 2009). Acetyl CoA is required for the acetyltransferase reactions involved in acetylation at lysine sites on newly synthesized proteins and these acetylation reactions can hinder ubiquitination and therefore prevent protein degradation (Sadoul et al., 2008). As such, the substantial reductions in brain acetyl CoA levels that occur after brain injury could have a negative impact on protein folding and stabilization. Misfolded proteins are targeted for destruction via endoplasmic reticulum associated degradation (ERAD). An example of this can be seen in knockout mice for the NAA-degrading enzyme ASPA. In ASPA-deficient mice, which cannot hydrolyze NAA into acetate and aspartate, there is a severe loss of several important myelin associated proteins including myelin basic protein and PLP/DM20 proteolipid proteins, combined with perinuclear retention of these proteins. These are indicators of impairment in protein trafficking in oligodendrocytes (Kumar et al., 2009). Oligodendrocytes are very susceptible to endoplasmic reticulum stress (ERS) associated with disruptions in protein synthesis and trafficking (Lin and Popko, 2009). Protein acetylation and deacetylation are also key regulatory mechanisms for controlling enzyme function in both the cytoplasm and in mitochondria (Guan and Xiong, 2010). It is quite possible that NAA-derived acetate is utilized to provide additional acetyl CoA for protein stabilization and enzyme regulation in oligodendrocytes after TBI-induced loss of acetyl CoA, but this possibility has not been investigated to date.

## POTENTIAL ROLE OF NAA IN DOPAMINERGIC NEUROTRANSMISSION

Recently, two laboratories have generated mice in which the gene for Asp-NAT (*Nat8l*) is knocked out (Furukawa-Hibi et al., 2012; Ariyannur et al., 2013). MRS analysis of the *Nat8l*± (heterozygous) mice indicated significant reductions (~30%) in forebrain NAA levels and the mice showed reduced vertical activity in open field tests (Ariyannur et al., 2013). Heterozygous *Nat8l*± mice also displayed increased responsiveness to methamphetamine. In studies of homozygous *Nat8l*-/- mice it was found that they exhibited reduced social interactions when in unfamiliar surroundings, as well as increased grooming and rearing in open field tests (Furukawa-Hibi et al., 2012; Ariyannur et al., 2013). Additionally, the mice had reduced levels of brain derived neurotrophic factor mRNA in the prefrontal cortex and hippocampus and decreased glial cell derived neurotrophic factor mRNA in the striatum and hippocampus. Earlier gene knockdown studies with antisense *Nat8l* oligonucleotides demonstrated that reduced Asp-NAT expression in the nucleus accumbens enhanced responsiveness to methamphetamine via increased dopamine release and reduced dopamine uptake (Niwa et al., 2007; Niwa and Nabeshima, 2011). These studies implicated Asp-NAT in driving tumor necrosis factor alpha expression which in turn reduced expression of brain derived and glial cell derived neurotrophic factors. The emerging view of NAA as either directly or indirectly involved in regulation of dopaminergic neurotransmission may open up new avenues of research and potential opportunities for therapeutic interventions into a variety of neurological and psychiatric disorders, including TBI which involves substantially reduced NAA levels.

Recent advancements including the cloning of the Asp-NAT gene now make it possible to selectively reduce NAA levels in key brain areas and to reverse the decreases by NAA supplementation with the hydrophobic methyl-ester of NAA to determine if the NAA reductions are associated with the pathologies observed in a wide variety of brain disorders and pathologies, including TBI. The RNAi knockdown method allows for selective reduction of NAA in specific brain regions of interest, which will mimic the reductions seen in various CNS disorders. Demonstration that NAA has critical roles in modulating dopaminergic neurotransmission would transform this field of research and possibly open up new therapeutic targets for a number of CNS disorders.

## NAA AND NAAG SYNTHESIS

*N*-Acetylaspartate is required for the synthesis of the dipeptide NAAG. NAAG is synthesized in neurons by the action of two recently discovered enzymes, designated NAAG synthetases (Becker et al., 2010; Collard et al., 2010). These two enzymes are encoded by genes for the ATP GRASP family proteins RIMKLA and RIMKLB. These enzymes are glutamate ligases that act to couple glutamate to an acceptor molecule. NAA is the primary acceptor for RIMKLA, whereas citrate is an additional acceptor for RIMKLB that also forms beta-citylglutamate. NAAG has been described as the most prevalent neuroactive peptide in the mammalian CNS (Neale et al., 2000). NAAG has been implicated in neurotransmitter release modulation (Adedoyin et al., 2010; Walder et al., 2012; Zuo et al., 2012; Romei et al., 2013) possibly via action at type 3 metabotropic glutamate receptors (Zhao et al., 2001; Neale, 2011). A number of studies have demonstrated that the concentration of NAA directly affects NAAG synthesis wherein higher concentrations NAA lead to increased NAAG synthesis (Gehl et al., 2004; Arun et al., 2006; Collard et al., 2010) and lower concentrations of NAA result in reduced NAAG synthesis (Ariyannur et al., 2013). Because NAA is a direct precursor for NAAG synthesis it is likely that reduced NAAG levels that are observed in experimental TBI (Vagnozzi et al., 2007) are due to reductions in the concentration of immediate precursor, NAA. Reduced NAAG levels after brain injury could have deleterious effects on neurotransmitter release modulation or other brain functions.

### NAAG CATABOLISM AND BRAIN INJURY

*N*-acetylaspartylglutamate is catabolized primarily by the enzyme glutamate carboxypeptidase II (GCPII, and also known as NAALADase) which hydrolyzes NAAG into NAA and glutamate (Blakely et al., 1988). GCPII is expressed on astrocytes (Berger et al., 1999) and has a high degree of similarity with prostate specific membrane antigen (Berger et al., 1999; Tiffany et al., 1999). Limiting NAAG hydrolysis with GCPII inhibitors has been found to be protective in brain injury models, and this is thought to occur by preventing glutamate release from NAAG, thus reducing post-injury excitotoxicity (Slusher et al., 1999; Vornov et al., 1999; Thomas et al., 2000). However, NAAG injected into the rat brain immediately before middle cerebral artery occlusion reduced infarct volume, and this effect was attenuated by co-treatment with a non-selective metabotropic glutamate receptor antagonist suggesting that NAAG is neuroprotective via binding to metabotropic glutamate receptors (Lu et al., 2000). Reducing NAAG hydrolysis with GCPII inhibitors in a rat model of TBI with secondary hypoxia was found to significantly reduce cell death in the hippocampus by maintaining NAAG levels and reducing glutamate release from NAAG (Gurkoff et al., 2013). Overall, the results point to intact NAAG as having neuroprotective effects through metabotropic glutamate receptors, and NAAG hydrolysis having excitotoxic effects through the action of the released glutamate on ionotropic glutamate receptors.

## FINAL THOUGHTS

*N*-Acetylaspartate is by far the most concentrated acetylated metabolite in the human brain, and its concentration is exquisitely responsive to brain injury. One possible explanation for the rapid and substantial drop in NAA levels immediately after injury is that NAA is providing local acetate as one of the mechanisms of response to depleted acetyl CoA that occurs due to injury-related energy depletion and metabolic depression. NAA synthesis requires the consumption of acetyl CoA, and the hydrolysis of NAA via ASPA generates local free acetate which can only be reconverted to acetyl CoA if either AceCS1 or AceCS2 are expressed at or near the site of NAA hydrolysis. Oligodendrocytes are one cell type in the brain that strongly express both ASPA and AceCS1 in their cytoplasm and nuclei, and therefore it seems likely that oligodendrocytes are a major site of acetyl CoA regeneration from NAA-derived acetate. The most parsimonious explanation for this arrangement is that NAA is acting as component of a neuron-oligodendrocyte metabolite trafficking system supporting oligodendrocyte metabolism during brain development, in the adult brain, and in response to brain injury. In addition, the strong expression of ASPA and AceCS1 in oligodendrocyte nuclei suggests that NAA-derived acetate is, in part, utilized to supply acetyl CoA for histone acetyltransferase enzymes required for increased gene transcription associated with injury-response mechanisms. NAA-derived acetate can also be used for myelin synthesis to repair myelin sheaths damaged during brain injury. In return, oligodendrocytes can supply neuronal axons with needed metabolites required for maintenance and repair.

Many gaps remain in our knowledge of the functional roles of NAA in the nervous system, and in other tissues such as BAT. Top among these is the final fate and distribution of NAA-derived acetate in the brain during development, and under different physiological and pathological conditions. It remains to be determined what proportion of NAA-derived acetate is directed to protein acetylation, lipid synthesis or energy derivation in the normal and injured brain. Other possible roles for NAA such as activation of second messenger systems, allosteric regulation of enzyme activity, or gene activation remain under-investigated and mostly unknown. Accumulating evidence links NAA to the regulation of dopamine release, but this requires further study to establish the connection and determine the underlying mechanisms. Evidence for a mitochondrial localization of Asp-NAT is mounting, but the mechanisms whereby Asp-NAT is targeted to mitochondrial membranes or matrix remain elusive due to a lack of an N-terminal mitochondrial targeting sequence. Additionally, it is unknown how much of a drop in NAA concentration *in vivo* is necessary to have an impact on NAAG synthesis, and therefore affect NAAG related functions. A less discussed aspect of NAA metabolism involves the fate of NAA-derived aspartate after ASPA-mediated hydrolysis. Is the resultant aspartate utilized primarily for protein synthesis or does it enter the TCA cycle as oxaloacetate for energy derivation? It is also possible some of the aspartate is recycled back to neurons for use there. Another area requiring further attention is the question of the identity of NAA transporters involved in moving NAA out of mitochondria, and moving NAA from neuronal axons to oligodendrocytes. Unraveling these uncertainties will be critical for understanding the complex nature of the roles played by NAA in the healthy and injured brain.

## Conflict of Interest Statement

The authors declare that the research was conducted in the absence of any commercial or financial relationships that could be construed as a potential conflict of interest.
